# High doses of oral folate and sublingual vitamin B12 in dialysis patients with hyperhomocysteinemia

**DOI:** 10.15171/jrip.2016.28

**Published:** 2016-08-06

**Authors:** Mitra Naseri, Gholam-Reza Sarvari, Mohammad Esmaeeli, Anoush Azarfar, Zahra Rasouli, Giti Moeenolroayaa, Shohre Jahanshahi, Simin Farhadi, Zohreh Heydari, Narges Sagheb-Taghipoor

**Affiliations:** Hemodialysis Section, Dr. Sheikh Children Hospital, Mashhad University of Medical Sciences, Mashhad, Iran

**Keywords:** Hyperhomocysteinemia, Folate, Vitamin B12, Dialysis

## Abstract

**Introduction:** Folic acid and vitamin B12, alone or in combination have been used to reduce homocysteine (Hcy) levels in dialysis patients.

**Objectives:** We aimed to assess the efficacy of high doses of oral folate and vitamin B12 in reducing plasma Hcy levels after a 12-week treatment.

**Patients and Methods:** Thirty-two dialysis patients aged 10-324 months screened for hyperhomocysteinuria. Then cases with hyperhomocysteinemia received oral folate 10 mg/day with sublingual methylcobalamin 1 mg/day for 12 weeks. In pre- and post-intervention phases plasma Hcy concentration, serum folate, and vitamin B12 levels were measured. Changes in plasma Hcy, serum folate, and vitamin B12 concentrations were analyzed by paired *t* tests, and *P* values < 0.05 were considered significant.

**Results:** Eighteen (56.2%) patients had hyperhomocysteinuria. Vitamin B12 and folate levels were normal or high in all cases. Two patients were lost due to transplant or irregular drugs consumption. Plasma Hcy levels were reduced in all, and reached normal values in 50%. A statistically significant differences between first Hcy levels with levels after intervention was found (95% CI, 5.1–8.9, *P* = 0.0001).

**Conclusion:** Oral folate 10 mg/day in combination with sublingual vitamin B12, 1 mg/day can be considered as a favorable treatment for hyperhomocysteinemia in dialysis patients.

**Implication for health policy/practice/research/medical education:**

In an interventional study in dialysis patients, high doses oral folate (10 mg/day) and sublingual vitamin B12 (1 mg/day) returned serum homocysteine (Hcy) levels to normal values in 50% and reduced levels in all. This combination therapy was safe and without serious short-term side effects.

## Introduction

Hyperhomocysteinemia, a modifiable, independent risk factor for coronary artery disease, stroke, and deep vein thrombosis (1-8), is frequently reported in chronic kidney diseases (CKD) (9), and is estimated to be present in 80%–90% of hemodialysis (HD) cases (10,11). A meta-analysis of genetic studies and prospective studies supported a causal association between homocysteine (Hcy) and cardiovascular disease (12). Atherosclerotic cardiovascular disease is the cause of death in 25%–60% of dialysis patients and also is a major cause of chronic morbidity.

Although the physiological basis for hyperhomocysteinemia in renal failure remains unclear (9), subclinical deficiency of folic acid and vitamin B12, two cofactors needed in the remethylation pathway (13,14), cause significant reductions in the clearance of Hcy by the kidney (9-13). Different doses of folic acid, vitamin B12, vitamin B6, and folinic acid alone or in combination have been used in dialysis cases to reduce or even normalize Hcy levels (8,9,14-20).

A 12-week intervention with high doses of oral folate (10 mg/day) and sublingual vitamin B12 (1 mg/day) was designed, comprising three separate phases to compare the changes in plasma Hcy concentration before and after intervention in children and young adults undergoing either HD or chronic ambulatory peritoneal dialysis (CAPD).

## Patients and Methods

Thirty-two dialysis patients who were supervised by the HD and CAPD departments of an academic pediatric health center were enrolled in the study. Patients were excluded if they currently used anti-folate or antiepileptic medications (carbamazepine and phenytoin), or if they did not wish to participate in the study.

The study was designed as three phases:

I: Phase 1 (week 0) or the screening phase for hyperhomocysteinemia (pre-intervention phase)
II: Phase 2 (week 0–12) or the intervention period
III: Phase 3 (after week 12) or post-intervention phase

### Screening phase

In this phase, blood samples were obtained during the fasting condition, during routine monthly samplings, and before dialysis (in HD cases) for measuring the plasma Hcy concentration, serum levels of folate, and vitamin B12.

### Intervention phase

The cases with hyperhomocysteinemia received oral folate 10 mg/day (2 tablets of 5 mg folic acid made by Rouz-Darou Company, Iran) in combination with sublingual methylcobalamin (biologically active form of vitamin B12 made by Bioclinic Natural Company, Canada) 1 mg/day for 12 weeks. The side effects of the drugs were explained for enrolled cases and asked participants to report any problem that could be related to the drugs.

### Post-intervention phase

In this step, blood samples were obtained to evaluate changes in plasma Hcy concentration, serum folate, and vitamin B12 levels after intervention. Plasma Hcy levels were measured by the Axis-Shield kit. The levels of 5–15 and >15 µmol/L were defined as normal and hyperhomocysteinemia, respectively. Serum vitamin B12 and folate levels were checked by the electrochemiluminescence immunoassay (ECLIA) method on Elecsys and Cobas e immunoassay analyzer (Roche Cobas e411 system). The sensitivity of the tests for vitamin B12 and folate was 30 ρg/mL and <20 ng/mL, respectively. Folate serum levels >20 ng/mL were only reported as high. Serum folate levels of 1.5–17 ng/mL were considered normal, and levels <1.5 ng/mL and >17 were defined as low (deficiency) and high, respectively. As for vitamin B12, levels <120 ρg/mL, 120–160 ρg/mL, 160–970 ρg/mL, and >970 ρg/mL were defined as deficient, borderline, normal, and high, respectively.

In our dialysis subjects, we routinely recommend low dose folate and vitamin B12 supplementation months before or immediately after placing on dialysis. The form of vitamin supplement that majority of our cases received was nephrovite tablet containing 0.5 mg folate, 6 µg vitamin B12, and 10 mg vitamin B6. Some patients received extra folate as folic acid tablets according to their physician recommendation. We did not recommend any changes in the doses of vitamin supplements that patients were receiving before the study. Table 1 summarizes the characteristics of enrolled patients.

**Table 1:** Characteristics of enrolled cases

**Patients’ characteristics**	
Age	10-324 (181.9 ± 82.5) months
Gender	15 (46.9%) girls and 17 ( 53.1%) boys
Modality of dialysis	21 (65.6%) HD and 12 (34.4%) CAPD subjects
Duration from onset of dialysis	1-153 (38.9 ± 38.5) months
Etiologies of CKD	Vesicoureteral reflux (VUR); 11 (34.4%) Neurogenic bladder; 7 (21.9%) Unknown; 7 (21.9%) Nephrotic syndrome; 4 (18.8%) Lupus nephropathy; 1 (3.1%) Familial juvenile nephronophtisis; 1 (3.1%) Autosomal recessive polycystic diseases; 1 (3.1%)
Dose of folate supplement before intervention	0.125-6 (1.5 ± 1.9) mg/day^a^, median 0.5 mg/day
Dose of vitamin B12 supplement before intervention	0-6 (4.2 ± 2.3) µg/day^b^, median 6 µg/day
History of renal transplantation	2 (6.3%) cases had received kidney transplantation in the past
Urine output	12 (37.5%) subjects were anuric
Hours of dialysis per week in HD cases	7-12 (10.2 ± 1.2)
Number of dialysis cycle per day in CAPD patients	3-6 (5±0.9)
Volume in each cycle (cc/kg) in CAPD patients	30-50 (37.7±6.4)
Duration of each cycle (hour) in CAPD patients	1-5 (3±0.96)

Abbreviations: HD, Hemodialysis; CAPD, continuous ambulatory peritoneal dialysis; CKD, Chronic kidney diseases. ^a^Twenty-one cases (65.6%) supplemented by 0.5 and 6 (18.7%) cases ≥5 mg/day. ^b^Twenty-four cases ( 75% ) supplemented by 6 µg/day vitamin B12 and 6 cases (18.7%) did not receive any vitamin B12 supplement.

### Ethical issues

1) The research followed the tenets of the Declaration of Helsinki; 2) informed consent was obtained, and they were free to leave the study at any time; and 3) the research was approved by the ethical committee of Mashhad University of Medical Sciences (Ethical code: IR.MUMS.REC.

1392.804).

### Statistical analysis

The normality of the variables were checked by one sample Kolmogorov-Smirnov test. The design of the study was pre- and post-test without a control group. We assumed that patients would follow the treatment (design of intention to treat). Descriptive statistics included mean ± standard deviation (SD) for continuous data, and percentage for categorical data. Chi-square and independent *t* tests were used for data analysis. Independent *t* and Mann–Whitney U tests were used for analysis of variables with normal (vitamin B12) and abnormal (folate) distribution. Changes in plasma Hcy, serum folate, and vitamin B12 concentrations in pre- and post-interventions were analyzed by paired *t* tests, and *P* values <0.05 were considered significant.

## Results

### Screening phase

Eighteen of the 32 (56.2%) patients had a plasma Hcy concentration >15 µmol/L. Majority of subjects with elevated Hcy concentration were in HD versus CAPD modality (16 versus 2 cases, respectively; *P* = 0.007), and the mean ± SD of age in cases with hyperhomocysteinemia was significantly higher than those with normal Hcy concentration (119.7 ± 60.1 and 230.2 ± 63.3 months, respectively; *P* = 0.001). Table 2 determines the results of plasma Hcy, serum folate, and vitamin B12 concentrations in the screening stage of the study. Vitamin B12 and folate levels were normal or high in all cases. There was no significant difference in serum vitamin B12 concentration between hyperhomocysteinemic subjects with those who had normal Hcy levels (*P* = 0.868).

**Table 2:** Plasma homocysteine, serum folate and vitamin B12 concentrations in enrolled cases

**Laboratory test**	**Minimum-maximum (mean ± SD) Median**
All enrolled cases	
Plasma homocysteine (µmol/L)	7-30 (16.5±5.7) 15.95
Serum folate (ng/mL)	15.8->20
Serum vitamin B12 (pg/mL)	296-1937 (813.5 ± 415) 740.7
Cases with hyperhomocysteinemia	
Plasma homocysteine (µmol/L)	15-30 (21 ± 4)
Serum folate (ng/mL)	8.15->20 ng/mL
Serum vitamin B12 (ρg/mL)	296-1730 (820.4 ± 416.4) ρg/mL
Cases with normal homocysteine levels	
Plasma homocysteine (µmol/L)	7-14 (11.4±2)
Serum folate (ng/mL)	9.1->20 ng/mL
Serum vitamin B12 (ρg/mL)	296-1730 (845.8 ± 428.5) ρg/ml

### Intervention phase

Eighteen cases enrolled in the intervention stage of the study. They received oral folate 10 mg and 1 mg sublingual vitamin B12 daily for 12 weeks. Two subjects complained of vomiting immediately after using sublingual vitamin B12, thus they were recommended to use the drug orally. Of 18 cases, one patient was lost to transplant, and one was lost due to irregular drugs consumption. No significant side effect in the intervention stage was reported by the cases. Any unpleasant drug reaction that required drug withdrawal was not reported during the intervention stage.

### Post-intervention phase

Plasma Hcy levels were reduced in all cases. Eight patients (50%) reached normal Hcy concentration. However, in 8 cases (50%), Hcy levels reduced after intervention, but did not reach a normal concentration.

**Figure 1:**
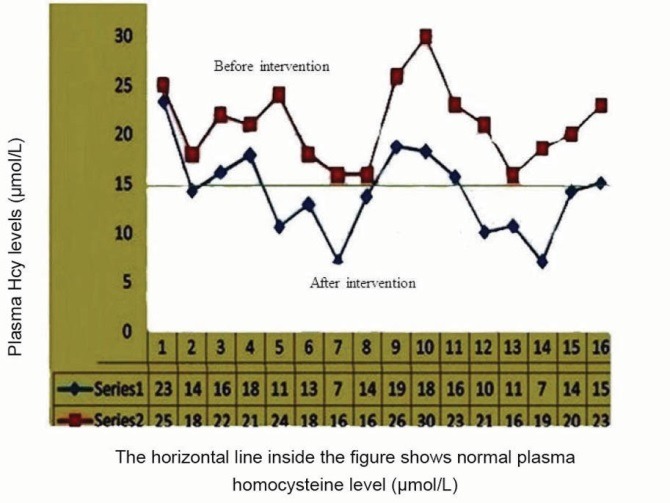
Plasma homocysteine changes after intervention

**Figure 2:**
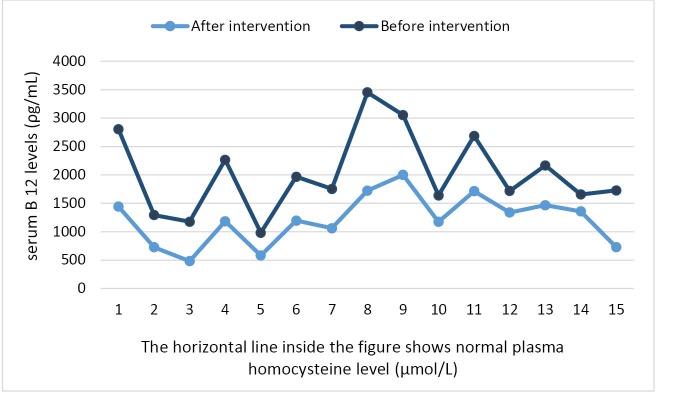
Serum vitamin B12 levels before and after intervention

**Figure 3:**
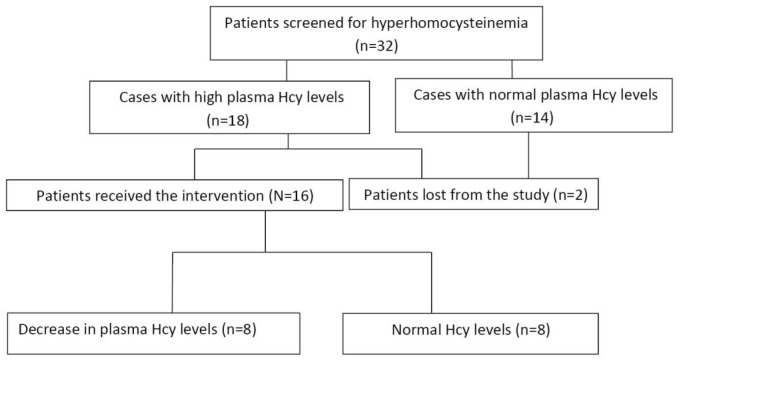
The flow of our participants in the study.

The plasma Hcy levels before intervention in enrolled cases were 15.9–30 (21.08 ± 4) µmol/L, which reached 7–23.3 (14.08 ± 4.4) µmol/L after intervention. The differences between first Hcy levels with levels after intervention (7.006 ± 3.5 µmol/L) were analyzed by paired *t* test (95% CI of the difference was 5.1–8.9, *P* = 0.0001, t = 7.85, df = 15). The intervention resulted in an 8–45.8% reduction in plasma Hcy levels. Serum vitamin B12 concentrations in cases before intervention were 296–1730 (778.9 ± 402.5) ρg/mL, while after intervention it reached 482–2000 (1182.6 ± 440.7) ρg/mL. Analysis of the differences between serum vitamin B12 levels before and after intervention by paired *t* test showed a significant increase in serum levels after intervention (95% CI: −629.14 and −178.16, *P* = 0.002, t = −38, and df = 15). Figures 1 and 2 present plasma Hcy and serum vitamin B12 changes after intervention. Before intervention, serum folate levels were normal in five (31%) and high in 11 (69%) cases with hyperhomocysteinemia, while the levels reached >20 ng/mL in all after intervention. It was interesting that in two subjects who used vitamin B12 as an oral route instead of sublingual, the Hcy concentration decreased drastically and reached a normal level in one case after intervention (levels before intervention were 22 and 18.6 µmol/L, which approached to be 16.1 and 7 µmol/L, respectively). Figure 3 illustrates the flow of our participants in the study.

## Discussion

To the best of our knowledge, this is the first study evaluating the response to high doses of oral folate combined with a sublingual form of vitamin B12 in dialysis patients for lowering the plasma Hcy levels. Based on literature reviewing that vitamin B12 has been used for treatment of hyperhomocysteinemia either as oral (10,16,20-22) or intravenous forms (15,23,24), no study has used the sublingual form of the drug in dialysis cases for treatment of hyperhomocysteinemia. We found a significant decrease in plasma Hcy levels by intervention, which supported the effectiveness of this treatment protocol for lowering the Hcy levels in dialysis cases.

As a 5 µmol/L increase in Hcy levels is associated with a relative risk of 1.5 (95% CI: 1.3–1.9) for ischemic stroke and 1.6 (95% CI: 1.4–1.7) and 1.8 (95% CI: 1.3–1.9) for cardiovascular heart diseases in men and women, respectively (25); thus, we can claim that our intervention resulted in a statistically significant decrease (7.006 ± 3.5 µmol/L, *P* = 0.0001) and clinically relevant decrease in plasma Hcy levels. Of course, it should be considered that in CKD patients there are multiple risk factors for cardiovascular heart diseases such as anemia and hypertension, which if not modified can interact with the beneficial effects of Hcy lowering interventions.

Supplementary folic acid in CKD cases effectively lower plasma Hcy levels, and it is recommended when the glomerular filtration rate (GFR) drops to <40 ml/min/1.73 m^2^, but the doses used are arbitrary. The amount of folic acid supplement, which is needed to lower Hcy levels, is more than that required to compensate for dialysis losses (26). Plasma Hcy lowering effects of B vitamins supplementation and high dose of folic acid have been shown in healthy populations (27,28). Folic acid 0.65–10 mg/day, alone or with vitamin B12 and/or B6, reduces the Hcy level by 25%–50%, both in healthy and hyperhomocysteinemic subjects (29). Numerous studies have reported that compared with cases with normal renal function in dialysis patients, higher doses of folic acid and vitamin B12 are needed to achieve a considerable reduction in Hcy levels (16,30).

The favorable effects of daily supplementation with folic acid, vitamin B6, vitamin B12, or a combination in reducing the Hcy levels has been reported in interventional studies (31). There are different controversies about the role of high doses of folate, vitamin B12, or their combination in the treatment of hyperhomocysteinemia. Manns et al (9) assessed Hcy lowering effects of 4-week daily oral Diavite (containing folate 1 mg, 6 µg vitamin B12, and 3 mg vitamin B6) in HD patients, and compared it with Diavite plus oral vitamin B12 at a dose of 1 mg/day for four weeks. In the third phase of the study, the subjects randomly received Diavite and 1 mg vitamin B12 daily plus placebo or folic acid 5 mg/day, or folic acid 20 mg/day, for four weeks. The study showed that the optimal oral treatment for hyperhomocysteinemia in HD patients is folic acid 1 mg and oral vitamin B12, 1 mg daily; Diavite for four weeks normalized the Hcy levels in 13.6% of patients.

Diavite tablet, which was used in the first phase of Manns et al study (9), has a very similar chemical component to nephrovite, which was recommended as routine vitamin supplements in our cases, except that the dose of folate was double of nephrovite (1 mg instead of 0.5 mg in each tablet). Majority of our cases who screened for hyperhomocysteinemia were receiving low dose folate (0.125–0.5 mg) and vitamin B12 (3–6 µg), daily for more than 4 weeks, but more than 50% of them had high plasma Hcy levels (≥15 µmol/L).

The NORVIT trial (32) identified a 27% reduction in serum Hcy concentration in patients receiving 0.8 mg folic acid plus 0.4 mg vitamin B12. The Hcy lowering effects of oral folate (2.5 up to 60 mg, daily) have been reported by different studies (33-35). Sunder-Plassmann et al (33) found that oral folate in doses of 15, 30, and 60 mg, daily for 4 weeks, have similar Hcy lowering effects. Azadibakhsh and colleagues (20) reported a significant decrease in plasma Hcy concentration after an 8-week treatment with oral folate 15 mg daily + oral vitamin B12, 1 mg daily. Tamadon et al (36) believed that folate 2 mg/day is sufficient to treat hyperhomocysteinemia in HD cases.

Gonin et al (21) compared the Hcy lowering effects of high doses of oral folate (30 and 60 mg/day) for 8 weeks alone or in combination with vitamin B6 and B12 supplements with placebo, and also intravenous folinic acid for 4 weeks with placebo. They found that none of these treatments resulted in a significant decrease in plasma Hcy levels compared with placebo. In contrast to the study of Gonin et al, Koyama et al (15) noted that high doses of oral folate (15 mg/day) with 0.5 mg intravenous vitamin B12 after any HD session (total of 9 injections) is an effective treatment for hyperhomocysteinemia.

The main differences between the study of Koyama et al and our study are that we recommended lower doses of folate (10 mg versus 15 mg daily), more prolonged duration of treatment (12 versus 3 weeks), and also different doses and different routes of vitamin B12 administration (1 mg in the form of sublingual tablet daily instead of intravenous injections of the vitamin after each HD session). The total doses of folate and vitamin B12, which were used in our study, were 2.5 and 20 times that of their study, respectively. They found that treatment with combined high doses of intravenous methylcobalamin and folic acid resulted in normalization of fasting Hcy levels in all 14 patients who participated in the study. Similar to our findings, the results of different studies confirmed Hcy lowering effects by a combination treatment with oral high doses of folate and vitamin B12 (16,24,37,38). A study in 11 HD patients with hyperhomocysteinemia revealed that 1 mg/day intravenous vitamin B12 for 4 weeks resulted in a 35% decrease in plasma Hcy concentration. The main limitation of our study comprised an absence of a control group. Since a limited number of patients were enrolled in our study, and just about half of them had high plasma Hcy levels, designing the study as a randomized clinical trial was not possible.

## Conclusion

Our study suggested that high doses of folate and vitamin B12 are needed to return the methylation pathway to its normal activity in addition to our treatment, which was a noninvasive, safe, and therapeutic method without serious short-term side effects, can be considered a favorable treatment for hyperhomocysteinemia in dialysis patients. However, the number of patients who enrolled in the intervention phases was very low. We recommend larger studies with adequate numbers of patients to confirm the results of this study.

## Limitations of this study

Small proportion of the patients was the main limitation of study.

## Acknowledgements

The authors would like to appreciate Dr. Varasteh and Mr. Akhlaghi (statistical consultant) for their advocated helps.

## Authors’ contribution

All authors contributed equally to the study.

## Conflicts of interest

The authors declared no competing interests.

## Ethical considerations

Ethical issues (including plagiarism, data fabrication, double publication) have been completely observed by the authors.

## Funding/Support

This study was extracted from thesis of Gholam-Reza Sarvari, fellowship of pediatric nephrology (Thesis# 920360). This study was funded by a research grant from the Research and Development Department of Mashhad University of Medical Sciences (Grant# 920360).
